# Supplementary material for: p53-armed oncolytic virotherapy induces abscopal effect in osteosarcoma by promoting immunogenic cell death
Source: Mol Ther Oncol. 2024 Jun 29;32(3):200845. doi: 10.1016/j.omton.2024.200845 (PMC11300929; doi:10.1016/j.omton.2024.200845)
Supplement: Document S1. Figures S1–S3 [file mmc1.pdf]

OMTON, Volume *32*

Supplemental information

p53-armed oncolytic virotherapy induces abscopal effect in osteosarcoma by promoting immunogenic cell death

Koji Demiya, Hiroshi Tazawa, Hiroya Kondo, Miho Kure, Yusuke Mochizuki, Tadashi Komatsubara, Aki Yoshida, Koji Uotani, Joe Hasei, Tomohiro Fujiwara, Toshiyuki Kunisada, Yasuo Urata, Shunsuke Kagawa, Toshifumi Ozaki, and Toshiyoshi Fujiwara

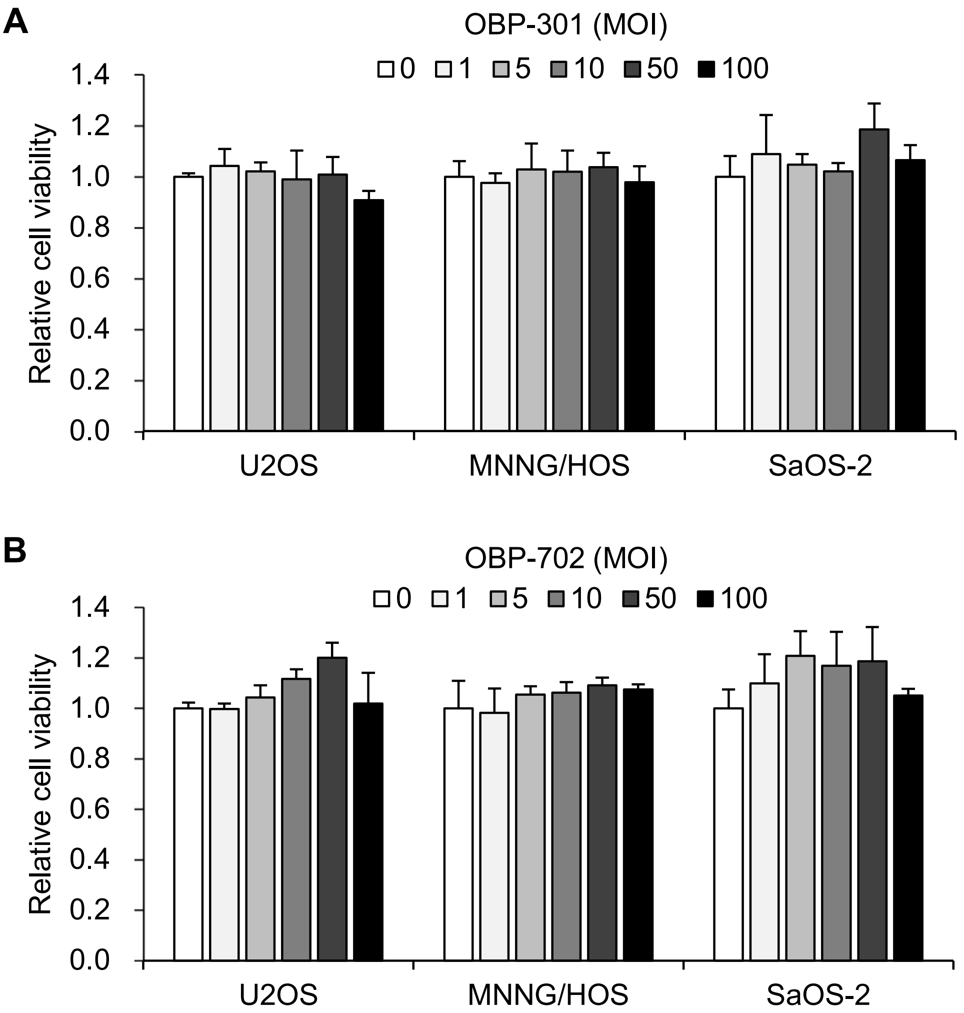

Figure S1. No cytopathic effect of OBP-301 and OBP-702 against human OS cells 24 h after infection. A,B U2OS, MNNG/HOS, and SaOS-2 cells were infected with OBP-301 (**A**) or OBP-702 (**B**) at the indicated multiplicity of infection (MOI), and cell viability was quantified 24 h after treatment using the XTT assay. Cell viability was calculated relative to that of the mock-infected group, which was set at 1.0. Cell viability data are expressed as mean values ±SD (n = 5).

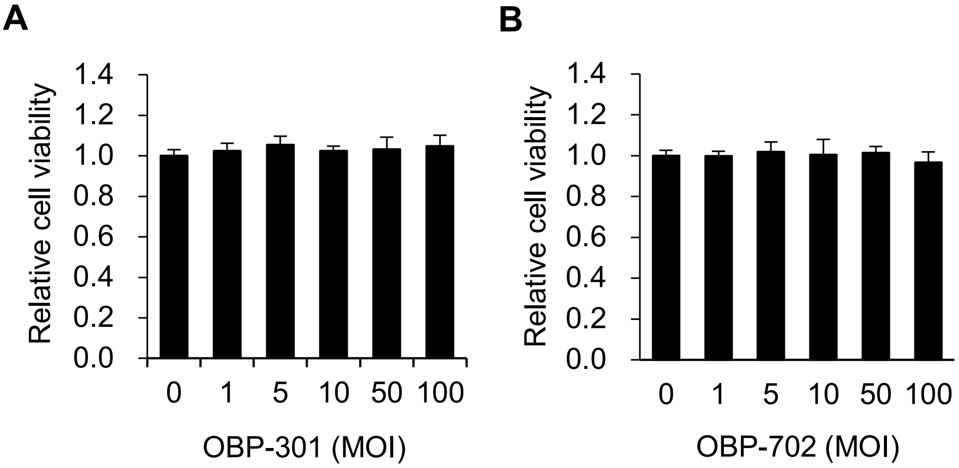

Figure S2. No cytopathic effect of OBP-301 and OBP-702 against murine OS cells 24 h after infection. A,B NHOS cells were infected with OBP-301 (**A**) or OBP-702 (**B**) at the indicated multiplicity of infection (MOI), and cell viability was quantified 24 h after treatment using the XTT assay. Cell viability was calculated relative to that of the mock-infected group, which was set at 1.0. Cell viability data are expressed as mean values ±SD (n = 5).

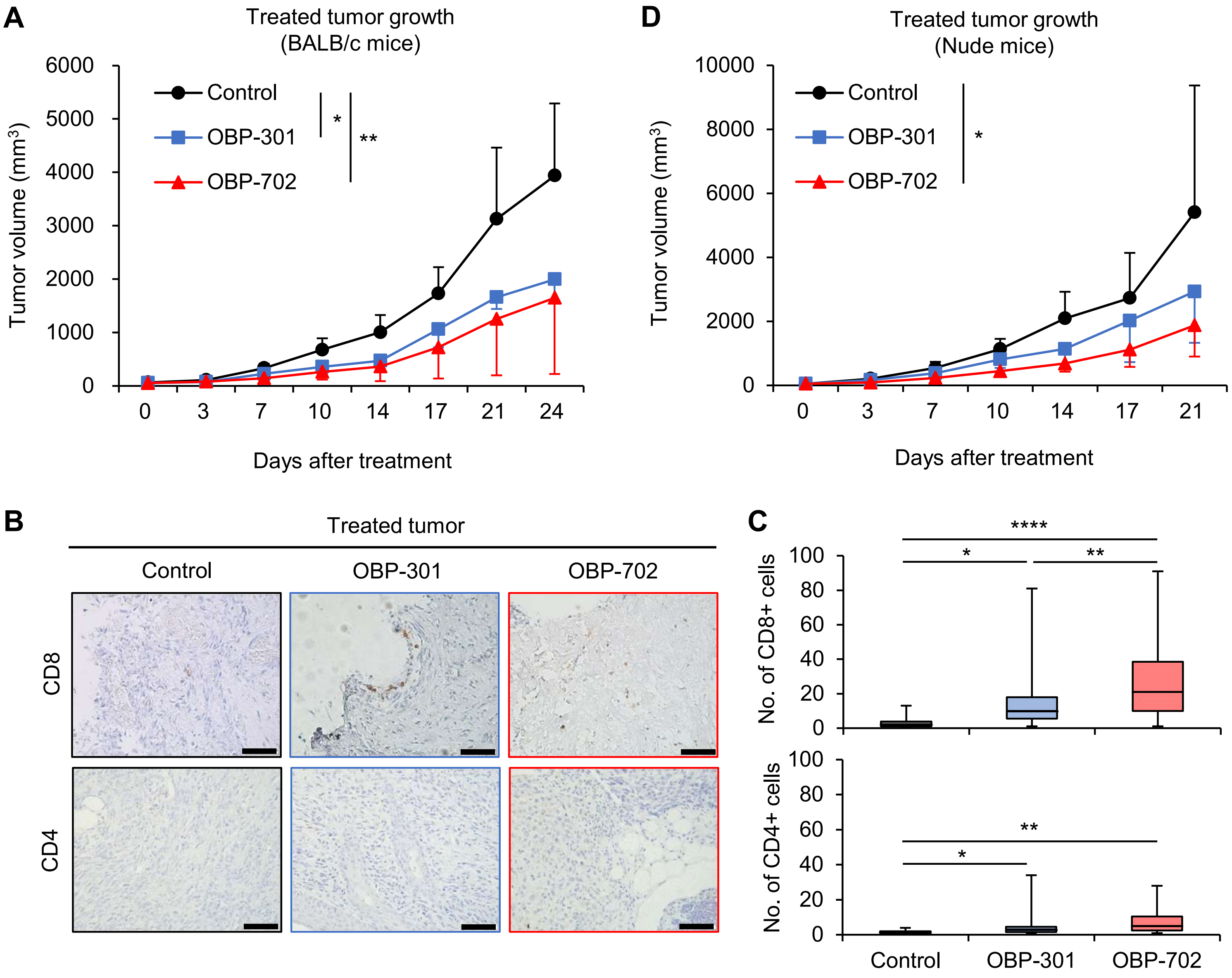

Figure S3. ***In vivo* antitumor effect of OBP-301 and OBP-702 in bilateral subcutaneous NHOS tumor models using immune-competent and immune-deficient mice. A** NHOS cells (2×10^6 cells/site) were inoculated into the bilateral flanks of immune-competent BALB/c mice. One side was intratumorally treated with PBS (n = 6), OBP-301 (blue arrows) (n = 7) or OBP-702 (red arrows) (n = 7) with 1×10^8 PFUs once a week for three cycles, and the other side was left untreated. The volume of NHOS tumors was monitored separately at the treated and untreated sites until day 24. **B** Representative photographs of immunohistochemical staining for CD8+ T cells and CD4+ T cells in treated tumors for each group. Scale bars, 100 µm. **C** The numbers of CD8+ T cells and CD4+ T cells were calculated from five different randomly selected fields. **D** The same experiment shown in **A** was performed using immune-deficient BALB/c-nu/nu nude mice, and tumor volume was monitored until day 21 (n = 8 in each group). Data are expressed as mean values ±SD. *$P < 0.05$, **$P < 0.01$, **** $P < 0.0001$.
