# Supplementary material for: p53-armed oncolytic virotherapy induces abscopal effect in osteosarcoma by promoting immunogenic cell death
Source: Mol Ther Oncol. 2024 Jun 29;32(3):200845. doi: 10.1016/j.omton.2024.200845 (PMC11300929; doi:10.1016/j.omton.2024.200845)
Supplement: Document S2. Article plus supplemental information [file mmc2.pdf]

Molecular Therapy
Oncology
Original Article

p53-armed oncolytic virotherapy induces abscopal effect in osteosarcoma by promoting immunogenic cell death

Koji Demiya,[1] Hiroshi Tazawa,[2,3] Hiroya Kondo,[1] Miho Kure,[1] Yusuke Mochizuki,[1] Tadashi Komatsubara,[1] Aki Yoshida,[1] Koji Uotani,[1] Joe Hasei,[1] Tomohiro Fujiwara,[1,4] Toshiyuki Kunisada,[1,5] Yasuo Urata,[6] Shunsuke Kagawa,[2,7] Toshifumi Ozaki,[1] and Toshiyoshi Fujiwara[2]

[1]Department of Orthopaedic Surgery, Okayama University Graduate School of Medicine, Dentistry and Pharmaceutical Sciences, Okayama 700-8558, Japan; [2]Department of Gastroenterological Surgery, Okayama University Graduate School of Medicine, Dentistry and Pharmaceutical Sciences, Okayama 700-8558, Japan; [3]Center for Innovative Clinical Medicine, Okayama University Hospital, Okayama 700-8558, Japan; [4]Department of Sports Medicine, Okayama University Graduate School of Medicine, Dentistry and Pharmaceutical Sciences, Okayama 700-8558, Japan; [5]Department of Medical Materials for Musculoskeletal Reconstruction, Okayama University Graduate School of Medicine, Dentistry and Pharmaceutical Sciences, Okayama 700-8558, Japan; [6]Oncolys BioPharma, Inc., Tokyo 105-0001, Japan; [7]Center for Clinical Oncology, Okayama University Hospital, Okayama 700-8558, Japan

Osteosarcoma (OS), the most frequent primary malignant tumor of bone in children and adolescents, is refractory to immune checkpoint inhibitors due to its poor antitumor immune response. Chemotherapy and virotherapy induce immunogenic cell death (ICD) and antitumor immune responses, leading to the abscopal effect in untreated tumors. We previously demonstrated the antitumor activity of the telomerase-specific replication-competent oncolytic adenoviruses OBP-301 and p53-armed OBP-702 in human OS cells. Here, we show the therapeutic potential of chemotherapeutic drugs (doxorubicin, cisplatin) and telomerase-specific oncolytic adenoviruses (OBP-301, p53-armed OBP-702) to induce ICD in human OS cells (U2OS, MNNG/HOS, SaOS-2) and murine OS cells (NHOS). OBP-702 induced more profound ICD via the secretion of adenosine triphosphate (ATP) and high-mobility group box protein B1 (HMGB1) compared with chemotherapy and OBP-301 in human OS cells. Murine NHOS cells were also more sensitive to OBP-702 than OBP-301. Subcutaneous NHOS tumor models demonstrated that intratumoral injection of OBP-702 significantly increased the tumor infiltration of cytotoxic CD8+ T cells and induced the abscopal effect against non-treated tumors compared with OBP-301. Our results suggest that OBP-702 is a promising antitumor reagent to induce ICD with secretion of ATP and HMGB1 and the abscopal effect against OS.

INTRODUCTION

Osteosarcoma (OS) is one of the most common primary malignant tumors of bone in children and young adults.[1,2] Despite current treatment strategies, including precision surgery and multi-agent chemotherapy, the prognosis of patients with OS has not improved over the past several decades.[3] Patients with OS with advanced primary tumors and distant metastases and those that are refractory to chemotherapy show poor prognosis.[2] Immune checkpoint inhibitors (ICIs) that target the programmed cell death 1 (PD-1)/programmed cell death ligand 1 (PD-L1) axis have been developed to improve the cancer immunity cycle.[4] The therapeutic efficacy of anti-PD-1 antibodies has been shown in certain cancer types[5]; however, clinical trials demonstrated that patients with sarcomas were refractory to PD-1 inhibitors showing only ∼5% partial response in patients with bone sarcomas.[6] Therefore, immune-stimulating therapies that promote the antitumor immune response against OS cells are needed for the treatment of patients with OS.

Immunogenic cell death (ICD) is a type of cell death that results in the release of damage-associated molecular patterns (DAMPs), including adenosine triphosphate (ATP) and high-mobility group box 1 (HMGB1), leading to the activation of antitumor immune responses.[7] Various chemotherapeutic drugs and radiation have been shown to induce ICD in malignant tumor cells.[8,9] Local treatment-mediated ICD induction subsequently promotes an antitumor immune response against not only treated tumors but also untreated tumors, a phenomenon known as the abscopal effect.[10] Local treatments that induce the abscopal effect represent a promising approach to target primary and metastatic OS tumors. By contrast, chemotherapy-mediated ICD induction promotes an antitumor immune response against metastatic relapse of malignant tumors. Although both cisplatin (CDDP) and doxorubicin (DOX) are the current first-line standard drugs in the treatment of OS tumors, low-dose DOX reportedly induces ICD in murine OS tumors, resulting in the promotion of an antitumor effect in dendritic cell vaccine therapy.[11]

Received 12 March 2024; accepted 28 June 2024;
https://doi.org/10.1016/j.omton.2024.200845.

Correspondence: Hiroshi Tazawa, Center for Innovative Clinical Medicine, Okayama University Hospital, Okayama 700-8558, Japan.
E-mail: htazawa@md.okayama-u.ac.jp

Published by Elsevier Inc. on behalf of The American Society of Gene and Cell Therapy.
This is an open access article under the CC BY license (http://creativecommons.org/licenses/by/4.0/).

Chemotherapies that induce ICD combined with immunotherapy represent a promising approach to prevent the recurrence and metastasis of OS tumors.

The tumor suppressor p53 protein plays a crucial role in the regulation of diverse cellular processes, including cell-cycle arrest, apoptosis, and autophagy.[12] Activation of p53 induced by the MDM2 inhibitor has been shown to induce ICD in p53-intact cancer cells.[13] However, the p53 gene is often inactivated due to somatic mutation in bone and soft-tissue sarcoma cells.[14] Recent report has shown that the restoration of p53 activity using the intracellular protein delivery platform Pos3Aa-p53 induces ICD against p53-null-type cancer cells.[15] Therefore, overexpression of exogeneous p53 protein would be useful strategy to induce ICD against OS cells independent of p53 status.

Oncolytic virotherapy has also been shown to induce ICD and promote antitumor immune responses via the secretion of ATP, HMGB1, and uric acid,[16–19] resulting in the enhancement of the antitumor efficacy of ICIs.[20] We developed a telomerase-specific replication-competent oncolytic adenovirus, OBP-301 (suratadenoturev), in which the *hTERT* (*human telomerase reverse transcriptase*) gene promoter drives the expression of the *E1A* and *E1B* genes.[21,22] The antitumor efficacy of OBP-301 in human OS cells was confirmed in monotherapy[23] or in combination with chemotherapy.[24,25] Moreover, we demonstrated that an Arginyl-glycyl-aspartic acid (RGD) fiber-modified OBP-301 variant (OBP-502) induces ICD and enhances the antitumor efficacy of PD-1 blockade in a syngeneic mouse model with murine OS tumors.[26] To enhance the therapeutic potential of OBP-301, we generated tumor-suppressor p53-armed OBP-702 and confirmed that it exhibits a stronger antitumor effect than OBP-301 against human OS cells.[27,28] Recently, we demonstrated that OBP-702-mediated p53 overexpression induces marked ICD that enhances the efficacy of PD-1 blockade against p53-wild-type and p53-mutant pancreatic cancer cells.[29] Therefore, we hypothesized that OBP-702 induces strong ICD and antitumor immune responses against OS cells independent of p53 status.

In the present study, we investigated the therapeutic potential of two chemotherapeutic agents (CDDP and DOX) and telomerase-specific oncolytic adenoviruses (OBP-301 and OBP-702) against human and murine OS cells. The *in vitro* cytopathic effect was assessed by analyzing cell viability using the 3′-{1-[(phenylamino)-carbonyl]-3,4-tetrazolium}-bis (4-methoxy-6-nitro) benzenesulfonic acid hydrate (XTT) assay. ICD induction was evaluated by analyzing the levels of extracellular ATP and HMGB1 secreted from therapy-treated OS cells. Therapy-induced apoptosis and autophagy were analyzed using western blotting. The *in vivo* antitumor efficacy and abscopal effect were evaluated using a subcutaneous murine OS tumor model.

RESULTS

CDDP and DOX induce ICD with release of HMGB1 in human OS cells

Chemotherapeutic agents such as DOX have been shown to induce ICD and activate the antitumor immune response via the secretion of DAMPs such as ATP and HMGB1.[9] To evaluate the therapeutic potentials of CDDP and DOX for inducing ICD in human OS cells, we used three human OS cell lines with different p53 statuses, U2OS (p53-wild type), MNNG/HOS (p53-mutant type), and SaOS-2 (p53-null type). Cell viability was assessed 24 h after treatment using the XTT assay. CDDP and DOX significantly suppressed the viability of all human OS cells in a dose-dependent manner (Figures 1A and 1B). Next, we investigated whether CDDP and DOX induce ICD by stimulating the secretion of ATP and HMGB1 in human OS cells. The levels of extracellular ATP and HMGB1 were analyzed using conditioned medium (CM) from U2OS, MNNG/HOS, and SaOS-2 cells at 24 h after treatment. At baseline, SaOS-2 cells secreted higher levels of ATP compared with U2OS and MNNG/HOS cells (Figures 1C and 1D). DOX significantly increased the secretion of ATP in U2OS cells, whereas MNNG/HOS and SaOS-2 cells showed significantly decreased secretion of ATP after treatment with CDDP or DOX (Figures 1C and 1D). By contrast, secretion of HMGB1 was significantly increased in U2OS and SaOS-2 cells after treatment with CDDP or DOX in a dose-dependent manner (Figures 1E and 1F). MNNG/HOS cells showed lower levels of HMGB1 release after treatment with DOX compared with U2OS and SaOS-2 cells (Figures 1E and 1F). These results suggest that CDDP and DOX have therapeutic potential to induce ICD in human OS cells by activating the release of HMGB1.

OBP-301 and OBP-702 induce ICD by stimulating secretion of ATP and HMGB1 in human OS cells

Oncolytic virotherapy has been shown to induce ICD and activate the antitumor immune response by stimulating the secretion of ATP and HMGB1.[19] To evaluate the therapeutic potential of the oncolytic adenoviruses OBP-301 and OBP-702 for inducing ICD in human OS cells, the viability of U2OS, MNNG/HOS, and SaOS-2 cells was assessed 24 h and 3 days after infection using the XTT assay. No decrease in the viability of human OS cells was observed 24 h after infection (Figures S1A and S1B). At 3 days after infection, OBP-301 and OBP-702 suppressed the viability of all human OS cell lines in a dose-dependent manner, and the cytopathic activity of OBP-702 was stronger than that of OBP-301 (Figures 2A and 2B). We next investigated whether OBP-301 and OBP-702 induce ICD in human OS cells. The levels of extracellular ATP and HMGB1 were analyzed using CM of human OS cells 24 h after infection. OBP-301 and OBP-702 significantly increased the secretion of ATP in all OS cell lines (Figures 2C and 2D). The level of extracellular ATP was approximately 20-fold higher with OBP-702-treated U2OS cells compared with OBP-301-treated cells (Figures 2C and 2D). By contrast, OBP-702 significantly increased the release of HMGB1 in all OS cell lines, whereas SaOS-2 cells showed an increased release of HMGB1 after OBP-301 treatment (Figures 2E and 2F). The level of extracellular HMGB1 was higher with OBP-702-treated OS cells compared with OBP-301-treated cells. However, oncolytic viruses induced lower levels of HMGB1 released from human OS cells compared with chemotherapeutic agents (Figures 2E and 2F). These results suggest that OBP-702 has stronger therapeutic potential than OBP-301 to induce ICD with secretion of ATP and HMGB1 by human OS cells.

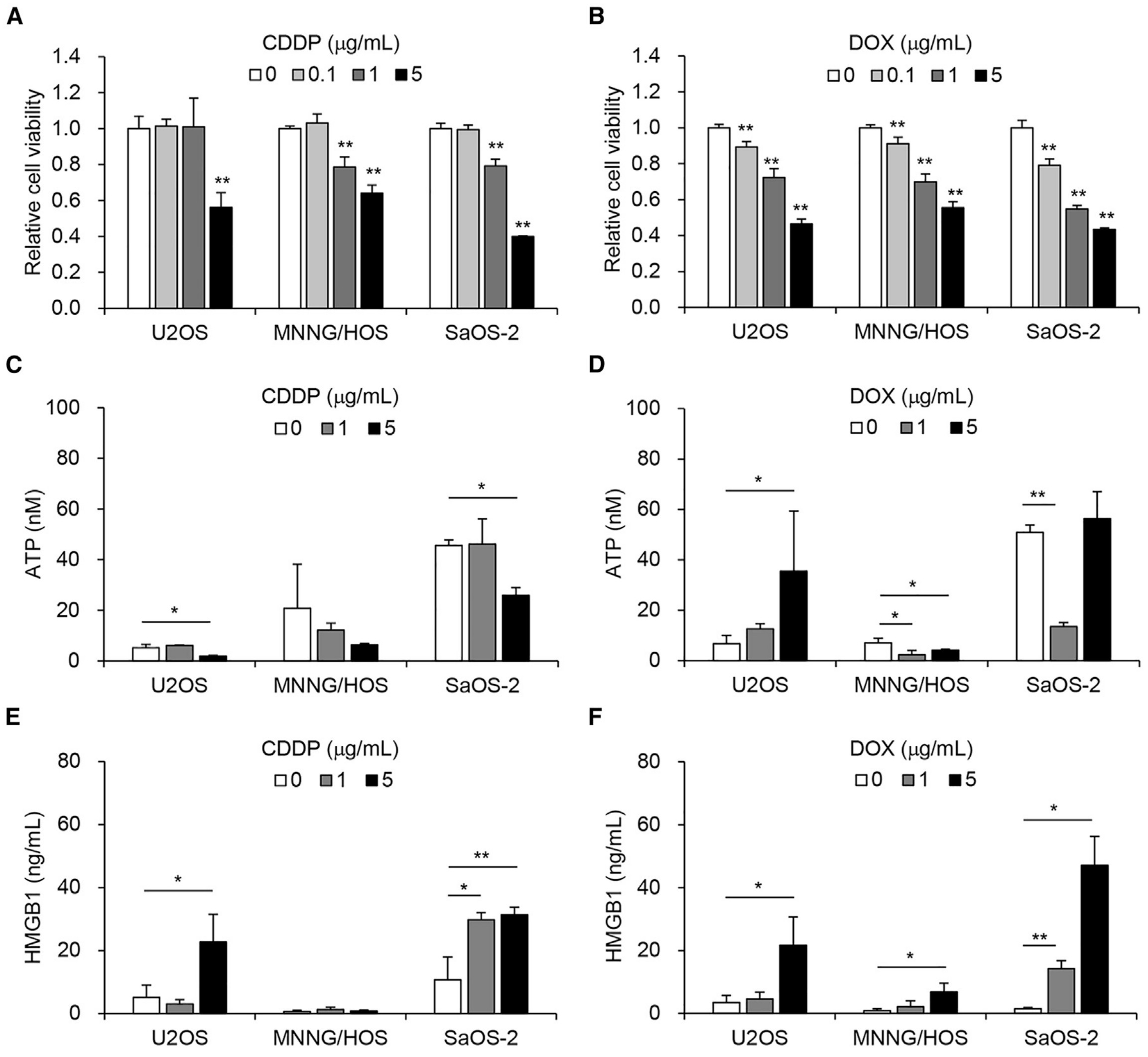

Figure 1. Cytopathic effect and ICD with secretion of ATP and HMGB1 induced by conventional chemotherapy in human OS cells

(A and B) U2OS, MNNG/HOS, and SaOS-2 cells were treated with cisplatin (CDDP) or doxorubicin (DOX) at the indicated doses, and cell viability was quantified 24 h after treatment using the XTT assay. Cell viability was calculated relative to that of the non-treated group, which was set at 1.0. Cell viability data are expressed as mean values ±SD (n = 5). (C–F) Cells were treated with CDDP or DOX (0, 1, and 5 μg/mL) for 24 h (n = 3). The levels of ATP and HMGB1 in supernatant were analyzed using the ENLITEN ATP assay (Promega, Madison, WI, USA) and HMGB1 ELISA kit (Shino-Test, Kanagawa, Japan), respectively. Data are expressed as mean values ±SD (n = 3 in each group; *$p < 0.05$ and **$p < 0.01$ [vs. 0 μg/mL]).

OBP-301 and OBP-702 induce ICD by stimulating secretion of ATP, HMGB1, and chemokines in murine OS cells

To evaluate the therapeutic potential of OBP-301 and OBP-702 against murine OS cells, the viability of murine NHOS cells was assessed 24 h and 3 days after infection using the XTT assay. No decrease in the viability of NHOS cells was observed 24 h after infection (Figures S2A and S2B). 3 days after infection, OBP-702 suppressed the viability of NHOS cells more strongly than OBP-301 (Figure 3A). Levels of extracellular ATP and HMGB1 were analyzed using the CM of NHOS cells 24 h after infection. OBP-301 significantly increased the secretion of HMGB1, but not ATP, whereas OBP-702 significantly increased the secretion of ATP and HMGB1 by NHOS cells (Figures 3B and 3C). Western blot analysis demonstrated that OBP-702 increased the expression of E1A, p53, and cleaved poly(ADP-ribose) polymerase (PARP) and decreased the expression of p62 in NHOS cells more strongly than OBP-301 (Figure 3D). These results suggest that OBP-702 has therapeutic potential to induce ICD with secretion of ATP and HMGB1 in murine OS cells more strongly than OBP-301.

We recently demonstrated that the RGD fiber-modified OBP-301 variant (OBP-502) induces the secretion of pro-inflammatory chemokines, CCL5 and CXCL10, from murine colorectal and pancreatic cancer cells.[30] The secretion of CXCL10 has been identified as an

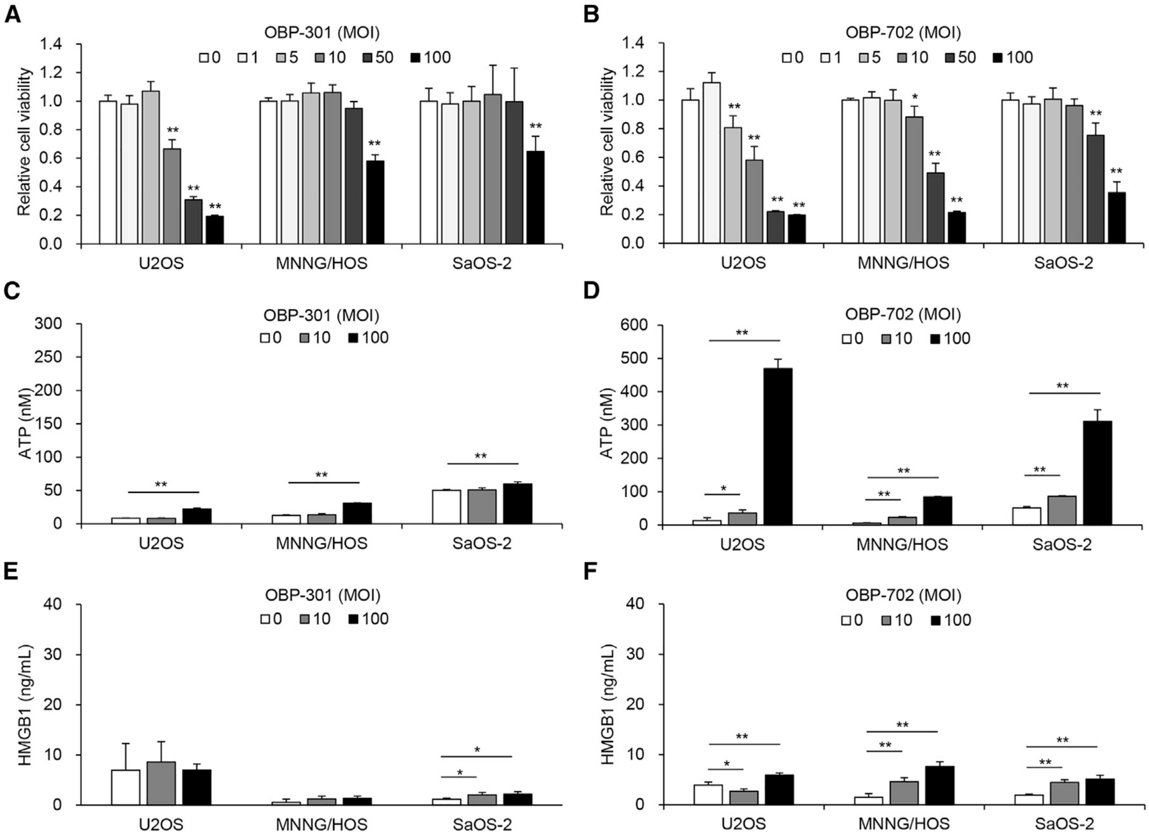

Figure 2. Cytotoxic effect and ICD induced in human OS cells by oncolytic adenoviruses with secretion of ATP and HMGB1

(A and B) U2OS, MNNG/HOS, and SaOS-2 cells were infected with OBP-301 or OBP-702 at the indicated MOI, and cell viability was quantified 24 h after treatment using the XTT assay. Cell viability was calculated relative to that of the mock-infected group on each day, which was set at 1.0. Cell viability data are expressed as mean values ±SD (n = 5). (C–F) Cells were treated with OBP-301 or OBP-702 (MOI 0, 10, and 100) for 24 h (n = 3). The levels of ATP and HMGB1 in the supernatant were analyzed using the ENLITEN ATP assay (Promega) and HMGB1 ELISA kit (Shino-Test), respectively. Data are expressed as mean values ± SD (n = 3 in each group; *p < 0.05 and **p < 0.01 [vs. MOI 0]).

ICD-related marker in association with the type I interferon response.[9] To evaluate the therapeutic potential of OBP-301 and OBP-702 for inducing the secretion of CCL5 and CXCL10 from murine OS cells, the levels of CCL5 and CXCL10 were analyzed using CM of NHOS cells 48 h after infection. ELISA demonstrated that OBP-301 and OBP-702 significantly increased the release of CCL5 and CXCL10 from NHOS cells (Figures 3E and 3F). These results suggest that OBP-301 and OBP-702 have the potential to induce the release of chemokines from murine OS cells.

OBP-301 and OBP-702 suppress the growth of murine NHOS tumors by enhancing the tumor infiltration of T cells

The *in vivo* antitumor effect of OBP-301 and OBP-702 against murine OS tumors was assessed using subcutaneous tumor models with murine NHOS cells. OBP-301 and OBP-702 were intratumorally injected in syngeneic BALB/c mice once a week for three cycles (Figure 4A). Both OBP-301 and OBP-702 significantly suppressed the growth of NHOS tumors, and the antitumor efficacy of OBP-702 was significantly higher than that of OBP-301 (Figures 4B and 4C). We next investigated whether OBP-301 and OBP-702 induce the accumulation of CD8+ T cells and CD4+ T cells in tumors. Immunohistochemistry analysis demonstrated that the numbers of CD8+ T cells and CD4+ T cells were significantly higher in virus-treated tumors compared with control tumors, and OBP-702 significantly increased the accumulation of T cells compared with OBP-301 (Figures 4D and 4E). These results suggest that OBP-301 and OBP-702 have therapeutic potential to reduce the growth of murine OS tumors by activating the tumor infiltration of T cells.

OBP-702 induces the abscopal effect by activating the antitumor immune response

Antitumor effects at untreated tumor sites are known as the abscopal effect in local treatments, including oncolytic virotherapy.[10] To assess the abscopal effect of OBP-301 and OBP-702, we used a syngeneic BALB/c mouse model involving bilateral subcutaneous NHOS tumors. One tumor side was intratumorally treated with OBP-301 or OBP-702 once a week for three cycles (Figure 5A). OBP-702 significantly suppressed tumor growth at both the treated and untreated

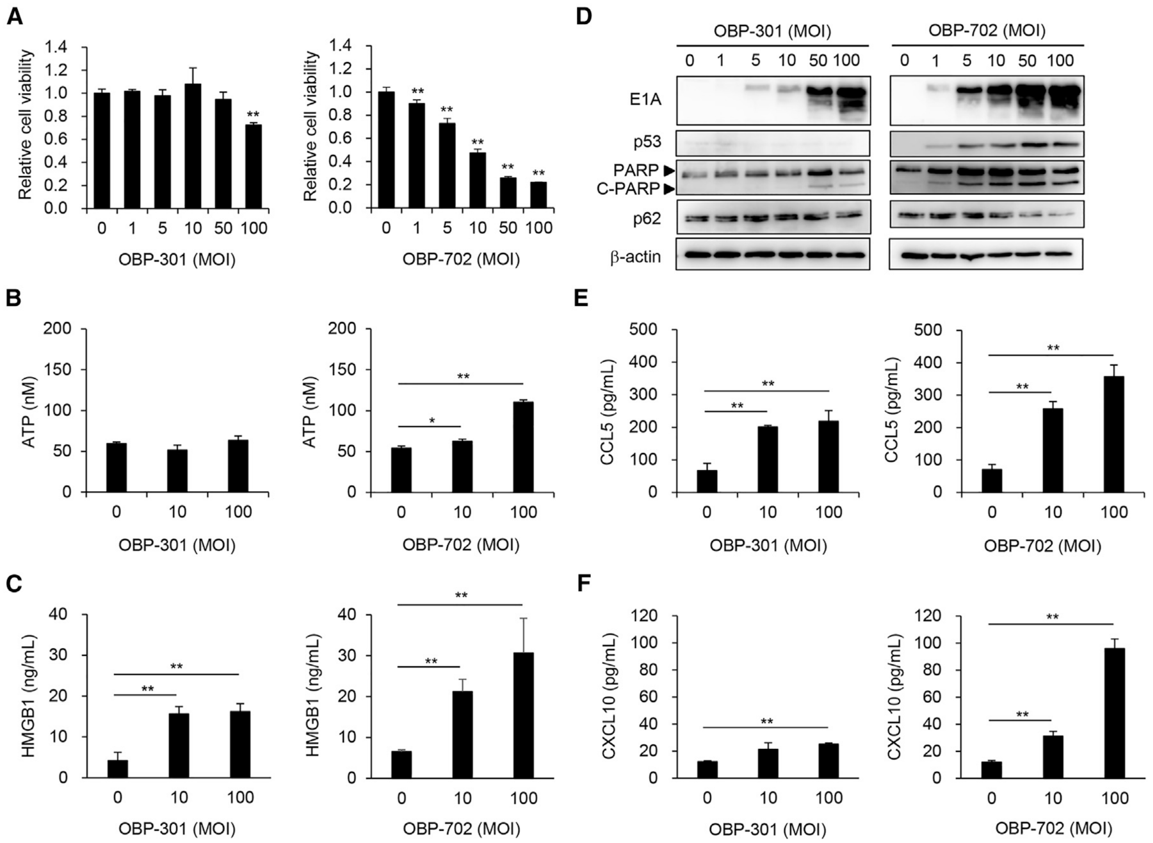

Figure 3. Cytotoxic effect and ICD with secretion of ATP and HMGB1 induced by oncolytic adenoviruses in murine OS cells

(A) NHOS cells were infected with OBP-301 or OBP-702 at the indicated MOI, and cell viability was quantified 24 h after treatment using the XTT assay. Cell viability was calculated relative to that of the mock-infected group on each day, which was set at 1.0. Cell viability data are expressed as mean values ±SD (n = 5). (B and C) Cells were treated with OBP-301 or OBP-702 (MOI 0, 10, and 100) for 24 h (n = 3). The levels of ATP and HMGB1 in the supernatant were analyzed using the ENLITEN ATP assay (Promega) and HMGB1 ELISA kit (Shino-Test), respectively. Data are expressed as mean values ± SD (n = 3 in each group; *$p < 0.05$ and **$p < 0.01$ [vs. MOI 0]). (D) NHOS cells were infected with OBP-301 or OBP-702 at the indicated MOIs for 72 h. Cell lysates were subjected to western blot analysis for E1A, p53, PARP, cleaved PARP (C-PARP), and p62. β-Actin was assayed as a loading control. (E and F) Supernatant of NHOS cells treated with OBP-301 or OBP-702 at the indicated MOIs for 72 h were used to analyze the amount of extracellular CCL5 (E) and CXCL10 (F) using ELISA. Data are expressed as mean values ± SD (n = 3 in each group; *$p < 0.05$ and **$p < 0.01$ [vs. MOI 0]).

sites compared with PBS and OBP-301 (Figures 5B and S3A). Immunohistochemistry analysis demonstrated that OBP-702 significantly increased the numbers of CD8+ T cells in treated and non-treated tumors compared with PBS and OBP-301, although the numbers of CD4+ T cells were significantly increased only in OBP-702-treated tumors (Figures 5C, 5D, S3B, and S3C). However, the abscopal effect of OBP-702 was diminished in immune-deficient nude mice, although the growth of treated tumors was reduced by OBP-702 (Figures 5E, 5F, and S3D). These results suggest that OBP-702 induces the abscopal effect by activating the antitumor immune response.

To investigate whether OBP-702 induces systemic antitumor immunity against murine OS cells, we conducted a rechallenge test using NHOS tumor model mice. NHOS cells were inoculated into the right flank of immune-competent BALB/c mice, and 7 days later, NHOS tumors were treated with OBP-702 or PBS every 2 days for 3 cycles. 3 days after the final treatment, pretreated NHOS tumors in the right flank were resected, and NHOS cells were reinoculated into the left flank of the same mice (Figure 6A). Pretreatment of the first NHOS tumors with OBP-702 significantly suppressed the growth of the second NHOS tumors (Figure 6B). These results suggest that OBP-702 induces systemic antitumor immunity against murine OS cells.

DISCUSSION

Immunogenic therapies that activate ICD and antitumor immune responses are needed to successfully treat OS tumors that are refractory to immunotherapy. In this study, we demonstrated that the telomerase-specific oncolytic adenoviruses OBP-301 and OBP-702 significantly induce stronger ICD against OS cells compared with conventional chemotherapy. OBP-301 and OBP-702 induced the release of higher levels of ATP and HMGB1 from human OS cells than the chemotherapeutic agents CDDP and DOX. OBP-702 exhibited a

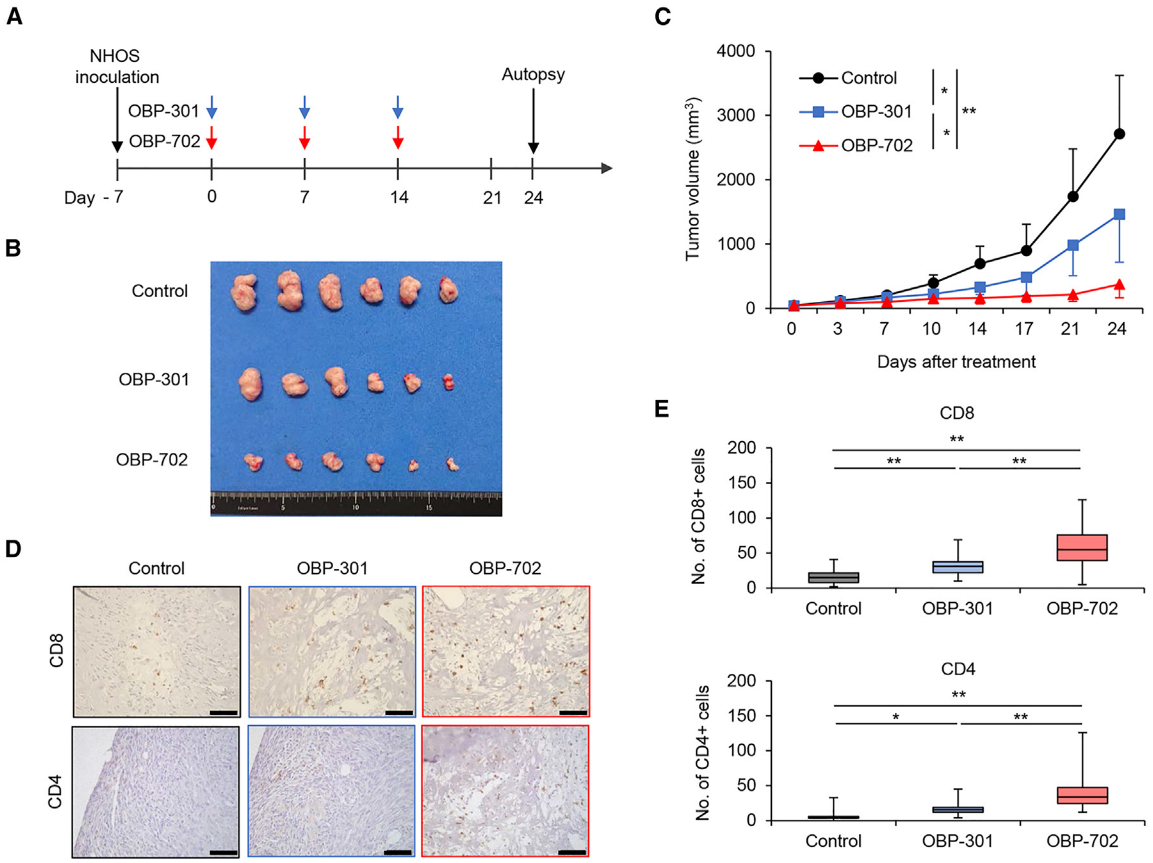

Figure 4. Recruitment of CD8+ T cells and antitumor effect of oncolytic adenoviruses against murine OS

(A–C) NHOS cells (2×10^6 cells/site) were inoculated into the flank of BALB/c mice. PBS (n = 6), OBP-301 (blue arrows) (n = 6), or OBP-702 (red arrows) (n = 6) was intratumorally injected along with 1×10^8 PFUs once a week for three cycles. (D) Representative photographs of immunohistochemical staining for CD8+ T cells and CD4+ T cells in each group. Scale bar, 100 μm. (E) The numbers of CD8+ T cells and CD4+ T cells were calculated from five different randomly selected fields. Data are expressed as mean values ±SD. *$p < 0.05$ and **$p < 0.01$.

marked antitumor effect against murine OS by inducing the tumor infiltration of CD8+ and CD4+ T cells. Moreover, OBP-702 significantly reduced tumor growth at untreated sites through the abscopal effect via the induction of systemic antitumor immunity. Thus, p53-armed oncolytic virotherapy is a promising antitumor strategy to induce the abscopal effect against OS via the induction of strong ICD and antitumor immune responses.

In the present study, the chemotherapeutic agents CDDP and DOX induced the secretion of HMGB1, but not ATP, in human OS cells (Figure 1). By contrast, the oncolytic adenoviruses OBP-301 and OBP-702 induced the secretion of both ATP and HMGB1 in human and murine OS cells (Figures 2 and 3). These findings suggest that the secretion of ATP can be induced more efficiently by oncolytic virotherapy than chemotherapy. With regard to the underlying mechanism of ATP secretion by cancer cells undergoing ICD, Martins et al. demonstrated that certain chemotherapeutic drugs, such as mitoxantrone and oxaliplatin, induce the secretion of ATP in human OS cells via caspase- and pannexin 1-dependent lysosomal exocytosis, which is associated with apoptosis and autophagy.[31,32] Many types of oncolytic viruses have been shown to play a significant role in the induction of ICD, apoptosis, and autophagy, leading to regulation of the immune system.[33] We previously demonstrated the therapeutic potential of OBP-702 to induce apoptosis- and autophagy-related death of human OS cells (MNNG/HOS, SaOS-2) by inducing p53 signaling pathways.[27] Recently, we demonstrated that p53-armed OBP-702 induces stronger ICD with secretion of ATP than non-armed OBP-301 against human and murine pancreatic cancer cells by activating p53 expression, apoptosis, and autophagy.[29] OBP-702 may induce the secretion of ATP from OS cells by activating apoptosis and autophagy.

Chemotherapy is used in the neoadjuvant/adjuvant setting for OS treatment. We previously demonstrated the antitumor effect of combination therapy with non-armed OBP-301 and CDDP/DOX against human OS cells by suppressing anti-apoptotic MCL1 expression.[24] Recently, we showed the antitumor effect of combination therapy with OBP-702 and DOX against DOX-resistant OS cells by

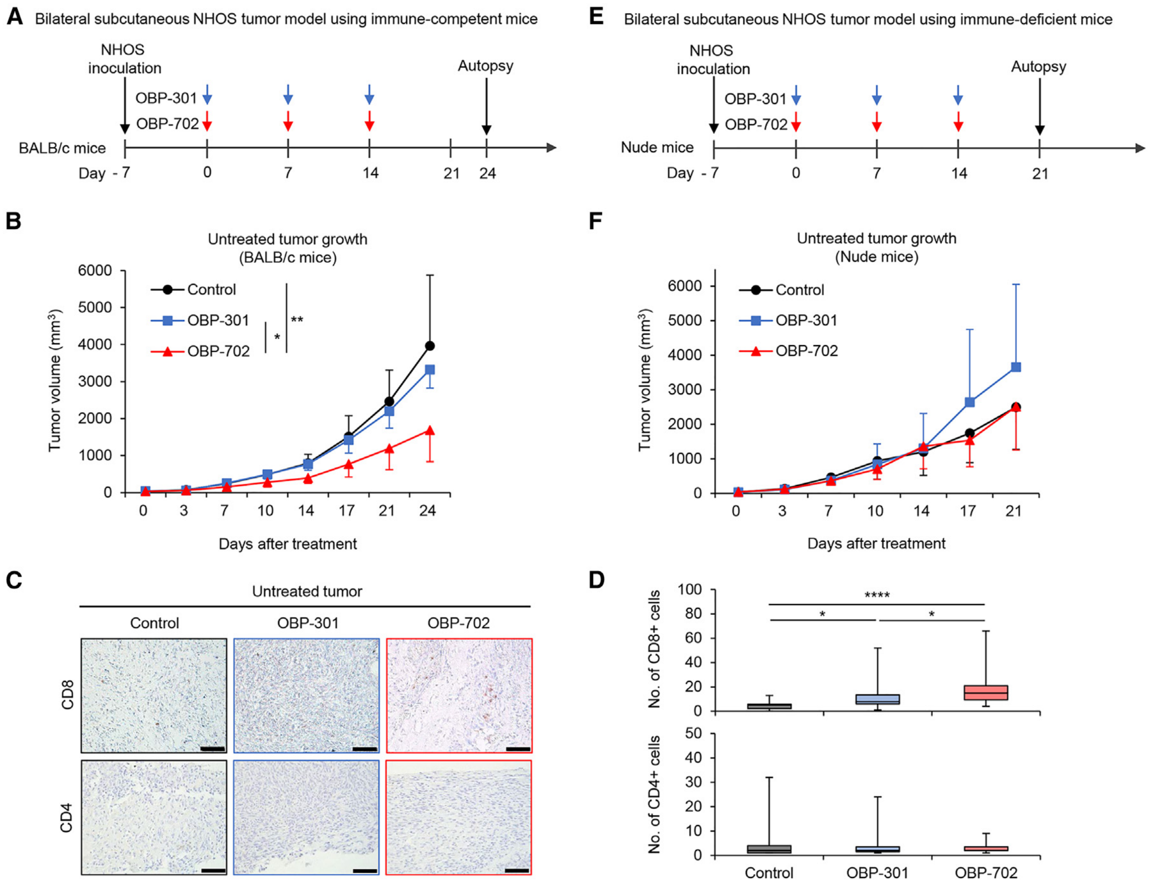

Figure 5. *In vivo* abscopal effect of OBP-702 in bilateral subcutaneous NHOS tumor model

(A and B) NHOS cells (2×10^6 cells/site) were inoculated into the bilateral flanks of immune-competent BALB/c mice. One side was intratumorally treated with PBS ($n = 6$), OBP-301 (blue arrows) ($n = 7$), or OBP-702 (red arrows) ($n = 7$) along with 1×10^8 PFUs once a week for three cycles, and the other side was left untreated. The volume of each NHOS tumor was monitored separately at the treated and untreated sites until day 24. (C) Representative photographs of immunohistochemical staining for CD8+ T cells and CD4+ T cells in untreated tumors for each group. Scale bar, 100 μm. (D) The numbers of CD8+ T cells and CD4+ T cells were calculated from five different randomly selected fields. (E and F) The same experiment shown in (A) was performed using immune-deficient BALB/c-nu/nu nude mice, and tumor volume was monitored until day 21 ($n = 8$ in each group). Data are expressed as mean values ± SD. $^*p < 0.05$, $^{**}p < 0.01$, and $^{****}p < 0.0001$.

suppressing drug-resistant MDR1 expression.[28] In this study, we observed that chemotherapeutic agents mainly induced the release of HMGB1 in human OS cells, whereas oncolytic viruses preferentially induced the release of ATP. As HMGB1 and ATP released from cancer cells have been shown to cooperatively promote the activation of dendritic cells in oncolytic virotherapy,[17] combination therapy may induce more profound ICD than monotherapy. Thus, further experiments are warranted to evaluate the therapeutic potential of combination therapy with oncolytic viruses and chemotherapy to induce ICD against OS cells.

OBP-702 elicited a stronger antitumor effect than OBP-301 by promoting tumor infiltration of CD8+ T cells in treated and untreated OS tumors (Figures 4 and 5). OBP-702-treated mice were more resistant to the development of second tumors compared with PBS-treated mice (Figure 6). These findings suggest that OBP-702 treatment induces systemic antitumor immunity to target OS cells. However, whether OBP-702-treated mice possess tumor-targeting cytotoxic T cells remains unclear. Regarding the underlying mechanism of the activation of tumor-infiltrating T cells, we observed that OBP-702 increased the release of CCL5 and CXCL10 from murine NHOS cells more strongly than OBP-301 (Figure 3). A significant relationship between the infiltration of CD8+ T cells and the expression of CCL5 and CXCL10 has been shown in Ewing sarcoma,[34] melanoma,[35] esophageal cancer,[36] and colorectal cancer.[37] Oncolytic adenoviruses expressing CCL5[38] or CXCL10[39] have been shown to induce the tumor infiltration of CD8+ T cells more strongly than non-armed viruses. Although the underlying mechanism of T cell accumulation in OBP-702-treated OS tissues remains unclear, induction of CCL5 and CXCL10 may be involved in the accumulation of T cells in OS tissues.

Clinical application of oncolytic viruses is expected as a novel antitumor modality for OS. Intratumoral injection of oncolytic viruses

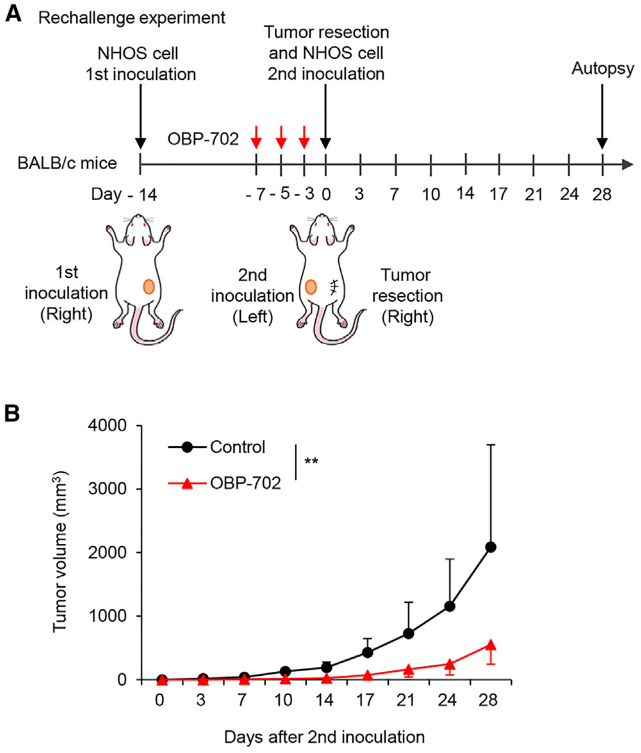

Figure 6. Rechallenge test of murine OS cells in OBP-702-treated syngeneic mice

(A and B) NHOS cells (2×10^6 cells/site) were inoculated into the right flank of BALB/c mice. 7 days later, right tumors were intratumorally treated with PBS (*n* = 5) or OBP-702 (red arrows) (*n* = 7) along with 1×10^8 PFUs every 2 days for three cycles. 3 days later, the right tumors were resected, and NHOS cells (2×10^6 cells/site) were further inoculated into the left flank of the same mice. The volume of the left tumors was monitored until day 28. Data are expressed as mean values ± SD. $^{**}p < 0.01$.

is needed to treat malignant tumors because neutralizing antibodies against the viruses impede the therapeutic potential of oncolytic viruses. In preclinical studies for orthotopic OS tumors, we previously demonstrated that intratumoral injection of OBP-702 induces a profound antitumor effect in human OS tumors in monotherapy[27] and combination therapy with zoledronic acid.[25] Martinez-Velez et al. also showed that intratumoral injection of oncolytic adenovirus Delta-24-ACT results in an antitumor effect in murine OS tumors.[40] In clinical studies for patients with OS, Stredy et al. demonstrated that computed-tomography-guided intratumoral injection of oncolytic herpes simplex virus 1 HSV1716 was safe and well tolerated by children and young adults with OS tumors.[41] These findings suggest that OS tumors are accessible for intratumoral injection of oncolytic viruses. Patients with OS may be suitable for treatment with oncolytic virotherapy.

Current clinical trials using ICIs have demonstrated that the prognosis of patients with OS is unfavorable.[42] A recent study that examined the immune-genomic landscape of OS tissues showed that OS tumors are cold tumors due to poor neoantigen expression and poor infiltration of immune cells.[43] Therefore, immune-activating therapies that promote the therapeutic potential of ICIs are needed. Oncolytic virotherapy has been shown to promote antitumor immune responses by activating ICD, improving the effectiveness of ICI treatment.[16,20] In this study, OBP-702 induced antitumor effects at treated sites by activating lytic cell death and the abscopal effect at untreated sites by activating ICD and subsequent antitumor immune responses in bilateral subcutaneous tumor models with NHOS cells (Figure 5). Recently, we demonstrated that RGD fiber-modified OBP-502 enhances the antitumor efficacy of PD-1 blockade against murine NHOS tumors by activating ICD and tumor infiltration of T cells.[26] More recently, OBP-702 was shown to promote the therapeutic potential of PD-L1 blockade against murine pancreatic cancer tumors by suppressing the tumor accumulation of immunosuppressive myeloid-derived suppressor cells.[44] Thus, further experiments are warranted to evaluate the therapeutic potential of combination therapy with OBP-702 and ICIs against cold OS tumors.

In conclusion, we demonstrated that the p53-armed telomerase-specific oncolytic adenovirus OBP-702 induces stronger ICD in human and murine OS cells by activating the secretion of DAMPs and proinflammatory chemokines, contributing to a marked increase in the infiltration of CD8+ T cells. Taken together, these data indicate that p53-armed oncolytic virotherapy is a novel therapeutic option for treating immunotherapy-refractory OS.

MATERIALS AND METHODS

Cell lines

The human OS cell line U2OS was obtained from the American Type Culture Collection (Manassas, VA, USA) and maintained in McCoy's 5a medium. The human OS cell line MNNG/HOS was purchased from DS Pharma Biomedical (Osaka, Japan) and maintained in Eagle's minimum essential medium containing 1% non-essential amino acids. The human OS cell line SaOS-2 was kindly provided by Dr. Satoru Kyo (Shimane University, Izumo, Japan) and maintained in Dulbecco's modified Eagle's medium. The murine OS cell line NHOS[45,46] was obtained from the Riken BioResource Research Center (Tsukuba, Ibaraki, Japan) and maintained in RPMI 1640 medium. All media were supplemented with 10% fetal bovine serum, 100 U/mL penicillin, and 100 μg/mL streptomycin. Cells were cultured for no longer than 5 months following resuscitation. All cells were maintained at 37°C in a humidified atmosphere with 5% CO_2.

Reagents

CDDP and DOX were purchased from Sigma-Aldrich (St. Louis, MO, USA).

Recombinant adenoviruses

The recombinant telomerase-specific replication-competent adenovirus OBP-301 (suratadenoturev), in which the promoter element of the *hTERT* gene drives the expression of *E1A* and *E1B* genes, was previously constructed and characterized.[21,22] For OBP-301-mediated induction of exogenous p53 gene expression, we generated

OBP-702, in which a human wild-type p53 gene expression cassette was inserted into the *E3* region of OBP-301.[47] Recombinant adenoviruses were purified using cesium chloride step gradients, and virus titers were determined by a plaque-forming assay using 293 cells; viruses were stored at −80°C.

Cell viability assay

Cells were seeded on 96-well plates at a density of 1×10^3 cells/well 24 h before treatment. Cells were then treated with CDDP or DOX at 0, 0.1, 1, or 5 μg/mL or infected with OBP-301 or OBP-702 at a multiplicity of infection (MOI) of 0, 1, 5, 10, 50, or 100 plaque-forming units (PFUs)/cell. Cell viability was determined 24 h after treatment with chemotherapeutic drugs or 3 days after virus infection using a Cell Proliferation Kit II (Roche Molecular Biochemicals, Indianapolis, IN, USA) according to the manufacturer's protocol.

DAMP analysis

Cells were seeded on 6-well plates at a density of 2×10^5 cells/well 24 h before treatment. Cells were then treated with CDDP or DOX at 0, 1, or 5 μg/mL or infected with OBP-301 or OBP-702 at an MOI of 0, 10, or 100 PFUs/cell. The culture supernatant was collected 48 h after treatment and analyzed using an ENLITEN ATP assay (Promega, Madison, WI, USA) and HMGB1 ELISA kit II (Shino-Test, Kanagawa, Japan) according to the manufacturers' protocols.

Western blot analysis

Cells were seeded in a 100 mm dish at a density of 2×10^5 cells/dish 24 h before treatment. Cells were then infected with OBP-301 or OBP-702 at an MOI of 0, 1, 5, 10, 50, or 100 PFUs/cell for 72 h. Whole-cell lysates were prepared in lysis buffer (50 mM Tris-HCl [pH 7.4], 150 mM NaCl, 1% Triton X-100) containing a protease inhibitor cocktail (Complete Mini; Roche, Indianapolis, IN, USA). Proteins were electrophoresed on 6%–15% sodium dodecyl sulfate-polyacrylamide gels and then transferred onto polyvinylidene difluoride membranes (Hybond-P; GE Healthcare, Buckinghamshire, UK). Blots were blocked by incubation with Blocking-One (Nacalai Tesque, Kyoto, Japan) at room temperature for 30 min. The primary antibodies used were mouse anti-Ad5 E1A monoclonal antibody (mAb) (BD PharMingen, Franklin Lakes, NJ, USA), rabbit anti-p53 mAb (Cell Signaling Technology, Beverly, MA, USA), rabbit anti-PARP polyclonal antibody (Cell Signaling Technology), mouse anti-p62 mAb (MBL, Nagoya, Japan), and mouse anti-β-actin mAb (Sigma-Aldrich, St. Louis, MO, USA). The secondary antibodies used were horseradish peroxidase-conjugated antibodies against rabbit immunoglobulin G (IgG; GE Healthcare) or mouse IgG (GE Healthcare). Immunoreactive bands on the blots were visualized using enhanced chemiluminescence substrates (ECL Plus; GE Healthcare).

In vivo subcutaneous NHOS tumor model

Animal experimental protocols were approved by the Ethics Review Committee for Animal Experimentation of the Okayama University School of Medicine (no. OKU-2018791). To evaluate the antitumor effect and antitumor immune response-stimulating effect of OBP-301 and OBP-702, NHOS cells (2×10^6 cells per site) were inoculated into the flank of 6-week-old female BALB/c mice (CLEA Japan, Tokyo, Japan). Palpable tumors developed within 7 days and were permitted to grow to approximately 5–6 mm in diameter. At that stage, a 20 μL volume of solution containing OBP-301 ($n = 6$) or OBP-702 ($n = 6$) at a dose of 1×10^8 PFUs or phosphate-buffered saline (PBS) ($n = 6$) was injected into the tumors once a week for three cycles. Tumor size was monitored twice a week by measuring tumor length and width using calipers. Tumor volume was calculated using the following formula: tumor volume (mm^3) = $L \times W^2 \times 0.5$, where L is the length and W is the width. 24 days after the first treatment, mice were sacrificed, and the tumors were harvested and fixed in formalin.

To evaluate the abscopal effect of OBP-301 and OBP-702, NHOS cells (2×10^6 cells per site) were inoculated into the bilateral flanks of 6-week-old female BALB/c mice. When tumors reached approximately 5–6 mm in diameter, a 20 μL volume of solution containing OBP-301 ($n = 7$) or OBP-702 ($n = 7$) at a dose of 1×10^8 PFU or PBS ($n = 6$) was injected into the tumors once a week for three cycles. To further analyze the role of antitumor immunity in the virus-induced abscopal effect, we performed the same experiments using 6-week-old female BALB/c-nu/nu nude mice ($n = 8$ in each group). Tumor size was monitored twice a week until 21 or 24 days after the first treatment.

To evaluate the therapeutic potential of OBP-702 for inducing systemic antitumor immunity, we performed rechallenge experiments. NHOS cells (2×10^6 cells per site) were inoculated into the right flank of 6-week-old female BALB/c mice. On day 7 after tumor inoculation, a 20 μL volume of solution containing OBP-702 ($n = 7$) at a dose of 1×10^8 PFUs or PBS ($n = 5$) was injected into the tumors every other day for three cycles. 3 days later, treated tumors were resected, and NHOS cells (2×10^6 cells per site) were reinoculated into the left flank of the same mice. Tumor size was monitored twice a week until 28 days after second tumor inoculation.

Immunohistochemistry

Paraffin-embedded tissue samples (4 μm) were deparaffinized in xylene and rehydrated in a graded ethanol series. After blocking endogenous peroxidases by incubation with 3% H_2O_2 for 10 min, the samples were boiled in citrate buffer or EDTA buffer for 14 min in a microwave oven for antigen retrieval. Samples were incubated with primary antibodies for 1 h at room temperature or overnight at 4°C and then with peroxidase-linked secondary antibody for 30 min at room temperature. After 3,3-diaminobenzidine staining for signal generation and counterstaining with Mayer's hematoxylin, samples were dehydrated and mounted onto coverslips. Antibodies against CD8 (eBioscience, San Diego, CA, USA) and CD4 (eBioscience) were used as primary antibodies. The number of cells expressing CD8, which is a marker of cytotoxic T lymphocytes, was calculated from five different randomly selected fields. All sections were analyzed under a light microscope.

Statistical analysis

Data are expressed as means ± SD. The significance of differences was assessed using the Student's t test. Differences between groups in animal experiments were assessed using one-way analysis of variance followed by Tukey's multiple-group comparison test. Statistical significance was defined as $p < 0.05$.

DATA AND CODE AVAILABILITY

All data generated or analyzed during this study are included in the main text or supplemental information. Further enquiries are directed to the corresponding author.

SUPPLEMENTAL INFORMATION

Supplemental information can be found online at https://doi.org/10.1016/j.omton.2024.200845.

ACKNOWLEDGMENTS

We thank Tomoko Sueishi, Yuko Hoshijima, and Tae Yamanishi for their excellent technical support. This study was supported in part by the Japan Agency for Medical Research and Development (AMED) (JP17ck0106285h0001 to Toshiyoshi Fujiwara) and Japan Society for the Promotion of Science (JSPS) KAKENHI grants (JP21K07219 to H.T., JP19K16835 to Y.M., JP18K15242 to T. Komatsubara, JP16K10862 to T. Kunisada, JP25293323 to T.O., and JP19H03731 to Toshiyoshi Fujiwara).

AUTHOR CONTRIBUTIONS

H.T., T.O., and Toshiyoshi Fujiwara developed the concept and designed research; K.D., H.K., M.K., Y.M., T. Komatsubara, A.Y., and K.U. performed research, acquired data, and analyzed and interpreted data; Y.U. supplied materials; K.D., H.T., and Toshiyoshi Fujiwara wrote and reviewed the manuscript; H.T., J.H., Tomohiro Fujiwara, T. Kunisada, S.K., T.O., and Toshiyoshi Fujiwara supervised the study.

DECLARATION OF INTERESTS

Y.U. is the president and CEO of Oncolys BioPharma, Inc. H.T. and Toshiyoshi Fujiwara are consultants of Oncolys BioPharma, Inc.

REFERENCES

1. Rojas, G.A., Hubbard, A.K., Diessner, B.J., Ribeiro, K.B., and Spector, L.G. (2021). International trends in incidence of osteosarcoma (1988-2012). Int. J. Cancer *149*, 1044–1053. https://doi.org/10.1002/ijc.33673.

2. Meltzer, P.S., and Helman, L.J. (2021). New Horizons in the Treatment of Osteosarcoma. N. Engl. J. Med. *385*, 2066–2076. https://doi.org/10.1056/NEJMra2103423.

3. Collins, M., Wilhelm, M., Conyers, R., Herschtal, A., Whelan, J., Bielack, S., Kager, L., Kuhne, T., Sydes, M., Gelderblom, H., et al. (2013). Benefits and adverse events in younger versus older patients receiving neoadjuvant chemotherapy for osteosarcoma: findings from a meta-analysis. J. Clin. Oncol. *31*, 2303–2312. https://doi.org/10.1200/JCO.2012.43.8598.

4. Topalian, S.L., Drake, C.G., and Pardoll, D.M. (2015). Immune checkpoint blockade: a common denominator approach to cancer therapy. Cancer Cell *27*, 450–461. https://doi.org/10.1016/j.ccell.2015.03.001.

5. Darvin, P., Toor, S.M., Sasidharan Nair, V., and Elkord, E. (2018). Immune checkpoint inhibitors: recent progress and potential biomarkers. Exp. Mol. Med. *50*, 1–11. https://doi.org/10.1038/s12276-018-0191-1.

6. Tawbi, H.A., Burgess, M., Bolejack, V., Van Tine, B.A., Schuetze, S.M., Hu, J., D'Angelo, S., Attia, S., Riedel, R.F., Priebat, D.A., et al. (2017). Pembrolizumab in advanced soft-tissue sarcoma and bone sarcoma (SARC028): a multicentre, two-cohort, single-arm, open-label, phase 2 trial. Lancet Oncol. *18*, 1493–1501. https://doi.org/10.1016/S1470-2045(17)30624-1.

7. Tesniere, A., Panaretakis, T., Kepp, O., Apetoh, L., Ghiringhelli, F., Zitvogel, L., and Kroemer, G. (2008). Molecular characteristics of immunogenic cancer cell death. Cell Death Differ. *15*, 3–12. https://doi.org/10.1038/sj.cdd.4402269.

8. Kroemer, G., Galluzzi, L., Kepp, O., and Zitvogel, L. (2013). Immunogenic cell death in cancer therapy. Annu. Rev. Immunol. *31*, 51–72. https://doi.org/10.1146/annurev-immunol-032712-100008.

9. Fucikova, J., Kepp, O., Kasikova, L., Petroni, G., Yamazaki, T., Liu, P., Zhao, L., Spisek, R., Kroemer, G., and Galluzzi, L. (2020). Detection of immunogenic cell death and its relevance for cancer therapy. Cell Death Dis. *11*, 1013. https://doi.org/10.1038/s41419-020-03221-2.

10. Janopaul-Naylor, J.R., Shen, Y., Qian, D.C., and Buchwald, Z.S. (2021). The Abscopal Effect: A Review of Pre-Clinical and Clinical Advances. Int. J. Mol. Sci. *22*, 11061. https://doi.org/10.3390/ijms222011061.

11. Kawano, M., Tanaka, K., Itonaga, I., Iwasaki, T., Miyazaki, M., Ikeda, S., and Tsumura, H. (2016). Dendritic cells combined with doxorubicin induces immunogenic cell death and exhibits antitumor effects for osteosarcoma. Oncol. Lett. *11*, 2169–2175. https://doi.org/10.3892/ol.2016.4175.

12. Vousden, K.H., and Prives, C. (2009). Blinded by the Light: The Growing Complexity of p53. Cell *137*, 413–431. https://doi.org/10.1016/j.cell.2009.04.037.

13. Guo, G., Yu, M., Xiao, W., Celis, E., and Cui, Y. (2017). Local Activation of p53 in the Tumor Microenvironment Overcomes Immune Suppression and Enhances Antitumor Immunity. Cancer Res. *77*, 2292–2305. https://doi.org/10.1158/0008-5472.CAN-16-2832.

14. Olivier, M., Hollstein, M., and Hainaut, P. (2010). TP53 mutations in human cancers: origins, consequences, and clinical use. Cold Spring Harbor Perspect. Biol. *2*, a001008. https://doi.org/10.1101/cshperspect.a001008.

15. Yang, Z., Sun, J.K., Lee, M.M., and Chan, M.K. (2022). Restoration of p53 activity via intracellular protein delivery sensitizes triple negative breast cancer to anti-PD-1 immunotherapy. J. Immunother. Cancer *10*, e005068. https://doi.org/10.1136/jitc-2022-005068.

16. Kaufman, H.L., Kohlhapp, F.J., and Zloza, A. (2015). Oncolytic viruses: a new class of immunotherapy drugs. Nat. Rev. Drug Discov. *14*, 642–662. https://doi.org/10.1038/nrd4663.

17. Tazawa, H., Kuroda, S., Hasei, J., Kagawa, S., and Fujiwara, T. (2017). Impact of Autophagy in Oncolytic Adenoviral Therapy for Cancer. Int. J. Mol. Sci. *18*, 1479. https://doi.org/10.3390/ijms18071479.

18. van Vloten, J.P., Workenhe, S.T., Wootton, S.K., Mossman, K.L., and Bridle, B.W. (2018). Critical Interactions between Immunogenic Cancer Cell Death, Oncolytic Viruses, and the Immune System Define the Rational Design of Combination Immunotherapies. J. Immunol. *200*, 450–458. https://doi.org/10.4049/jimmunol.1701021.

19. Palanivelu, L., Liu, C.H., and Lin, L.T. (2022). Immunogenic cell death: The cornerstone of oncolytic viro-immunotherapy. Front. Immunol. *13*, 1038226. https://doi.org/10.3389/fimmu.2022.1038226.

20. Bommareddy, P.K., Shettigar, M., and Kaufman, H.L. (2018). Integrating oncolytic viruses in combination cancer immunotherapy. Nat. Rev. Immunol. *18*, 498–513. https://doi.org/10.1038/s41577-018-0014-6.

21. Kawashima, T., Kagawa, S., Kobayashi, N., Shirakiya, Y., Umeoka, T., Teraishi, F., Taki, M., Kyo, S., Tanaka, N., and Fujiwara, T. (2004). Telomerase-specific replication-selective virotherapy for human cancer. Clin. Cancer Res. *10*, 285–292. https://doi.org/10.1158/1078-0432.ccr-1075-3.

22. Hashimoto, Y., Watanabe, Y., Shirakiya, Y., Uno, F., Kagawa, S., Kawamura, H., Nagai, K., Tanaka, N., Kumon, H., Urata, Y., and Fujiwara, T. (2008). Establishment of biological and pharmacokinetic assays of telomerase-specific

replication-selective adenovirus. Cancer Sci. *99*, 385–390. https://doi.org/10.1111/j.1349-7006.2007.00665.x.

23. Sasaki, T., Tazawa, H., Hasei, J., Kunisada, T., Yoshida, A., Hashimoto, Y., Yano, S., Yoshida, R., Uno, F., Kagawa, S., et al. (2011). Preclinical evaluation of telomerase-specific oncolytic virotherapy for human bone and soft tissue sarcomas. Clin. Cancer Res. *17*, 1828–1838. https://doi.org/10.1158/1078-0432.CCR-10-2066.

24. Osaki, S., Tazawa, H., Hasei, J., Yamakawa, Y., Omori, T., Sugiu, K., Komatsubara, T., Fujiwara, T., Sasaki, T., Kunisada, T., et al. (2016). Ablation of MCL1 expression by virally induced microRNA-29 reverses chemoresistance in human osteosarcomas. Sci. Rep. *6*, 28953. https://doi.org/10.1038/srep28953.

25. Yamakawa, Y., Tazawa, H., Hasei, J., Osaki, S., Omori, T., Sugiu, K., Komatsubara, T., Uotani, K., Fujiwara, T., Yoshida, A., et al. (2017). Role of zoledronic acid in oncolytic virotherapy: Promotion of antitumor effect and prevention of bone destruction. Cancer Sci. *108*, 1870–1880. https://doi.org/10.1111/cas.13316.

26. Mochizuki, Y., Tazawa, H., Demiya, K., Kure, M., Kondo, H., Komatsubara, T., Sugiu, K., Hasei, J., Yoshida, A., Kunisada, T., et al. (2021). Telomerase-specific oncolytic immunotherapy for promoting efficacy of PD-1 blockade in osteosarcoma. Cancer Immunol. Immunother. *70*, 1405–1417. https://doi.org/10.1007/s00262-020-02774-7.

27. Hasei, J., Sasaki, T., Tazawa, H., Osaki, S., Yamakawa, Y., Kunisada, T., Yoshida, A., Hashimoto, Y., Onishi, T., Uno, F., et al. (2013). Dual programmed cell death pathways induced by p53 transactivation overcome resistance to oncolytic adenovirus in human osteosarcoma cells. Mol. Cancer Ther. *12*, 314–325. https://doi.org/10.1158/1535-7163.MCT-12-0869.

28. Sugiu, K., Tazawa, H., Hasei, J., Yamakawa, Y., Omori, T., Komatsubara, T., Mochizuki, Y., Kondo, H., Osaki, S., Fujiwara, T., et al. (2021). Oncolytic virotherapy reverses chemoresistance in osteosarcoma by suppressing MDR1 expression. Cancer Chemother. Pharmacol. *88*, 513–524. https://doi.org/10.1007/s00280-021-04310-5.

29. Araki, H., Tazawa, H., Kanaya, N., Kajiwara, Y., Yamada, M., Hashimoto, M., Kikuchi, S., Kuroda, S., Yoshida, R., Umeda, Y., et al. (2022). Oncolytic virus-mediated p53 overexpression promotes immunogenic cell death and efficacy of PD-1 blockade in pancreatic cancer. Mol. Ther. Oncolytics *27*, 3–13. https://doi.org/10.1016/j.omto.2022.09.003.

30. Kanaya, N., Kuroda, S., Kakiuchi, Y., Kumon, K., Tsumura, T., Hashimoto, M., Morihiro, T., Kubota, T., Aoyama, K., Kikuchi, S., et al. (2020). Immune Modulation by Telomerase-Specific Oncolytic Adenovirus Synergistically Enhances Antitumor Efficacy with Anti-PD1 Antibody. Mol. Ther. *28*, 794–804. https://doi.org/10.1016/j.ymthe.2020.01.003.

31. Martins, I., Wang, Y., Michaud, M., Ma, Y., Sukkurwala, A.Q., Shen, S., Kepp, O., Metivier, D., Galluzzi, L., Perfettini, J.L., et al. (2014). Molecular mechanisms of ATP secretion during immunogenic cell death. Cell Death Differ. *21*, 79–91. https://doi.org/10.1038/cdd.2013.75.

32. Wang, Y., Martins, I., Ma, Y., Kepp, O., Galluzzi, L., and Kroemer, G. (2013). Autophagy-dependent ATP release from dying cells via lysosomal exocytosis. Autophagy *9*, 1624–1625. https://doi.org/10.4161/auto.25873.

33. Wu, Y.Y., Sun, T.K., Chen, M.S., Munir, M., and Liu, H.J. (2023). Oncolytic viruses-modulated immunogenic cell death, apoptosis and autophagy linking to virotherapy and cancer immune response. Front. Cell. Infect. Microbiol. *13*, 1142172. https://doi.org/10.3389/fcimb.2023.1142172.

34. Berghuis, D., Santos, S.J., Baelde, H.J., Taminiau, A.H., Egeler, R.M., Schilham, M.W., Hogendoorn, P.C., and Lankester, A.C. (2011). Pro-inflammatory chemokine-chemokine receptor interactions within the Ewing sarcoma microenvironment determine CD8(+) T-lymphocyte infiltration and affect tumour progression. J. Pathol. *223*, 347–357. https://doi.org/10.1002/path.2819.

35. Harlin, H., Meng, Y., Peterson, A.C., Zha, Y., Tretiakova, M., Slingluff, C., McKee, M., and Gajewski, T.F. (2009). Chemokine expression in melanoma metastases associated with CD8+ T-cell recruitment. Cancer Res. *69*, 3077–3085. https://doi.org/10.1158/0008-5472.CAN-08-2281.

36. Liu, J., Li, F., Ping, Y., Wang, L., Chen, X., Wang, D., Cao, L., Zhao, S., Li, B., Kalinski, P., et al. (2015). Local production of the chemokines CCL5 and CXCL10 attracts CD8+ T lymphocytes into esophageal squamous cell carcinoma. Oncotarget *6*, 24978–24989. https://doi.org/10.18632/oncotarget.4617.

37. Zumwalt, T.J., Arnold, M., Goel, A., and Boland, C.R. (2015). Active secretion of CXCL10 and CCL5 from colorectal cancer microenvironments associates with GranzymeB+ CD8+ T-cell infiltration. Oncotarget *6*, 2981–2991. https://doi.org/10.18632/oncotarget.3205.

38. Lapteva, N., Aldrich, M., Weksberg, D., Rollins, L., Goltsova, T., Chen, S.Y., and Huang, X.F. (2009). Targeting the intratumoral dendritic cells by the oncolytic adenoviral vaccine expressing RANTES elicits potent antitumor immunity. J. Immunother. *32*, 145–156. https://doi.org/10.1097/CJI.0b013e318193d31e.

39. Li, X., Lu, M., Yuan, M., Ye, J., Zhang, W., Xu, L., Wu, X., Hui, B., Yang, Y., Wei, B., et al. (2022). CXCL10-armed oncolytic adenovirus promotes tumor-infiltrating T-cell chemotaxis to enhance anti-PD-1 therapy. OncoImmunology *11*, 2118210. https://doi.org/10.1080/2162402X.2022.2118210.

40. Martinez-Velez, N., Laspidea, V., Zalacain, M., Labiano, S., Garcia-Moure, M., Puigdelloses, M., Marrodan, L., Gonzalez-Huarriz, M., Herrador, G., de la Nava, D., et al. (2022). Local Treatment of a Pediatric Osteosarcoma Model with a 4-1BBL Armed Oncolytic Adenovirus Results in an Antitumor Effect and Leads to Immune Memory. Mol. Cancer Ther. *21*, 471–480. https://doi.org/10.1158/1535-7163.MCT-21-0565.

41. Streby, K.A., Geller, J.I., Currier, M.A., Warren, P.S., Racadio, J.M., Towbin, A.J., Vaughan, M.R., Triplet, M., Ott-Napier, K., Dishman, D.J., et al. (2017). Intratumoral Injection of HSV1716, an Oncolytic Herpes Virus, Is Safe and Shows Evidence of Immune Response and Viral Replication in Young Cancer Patients. Clin. Cancer Res. *23*, 3566–3574. https://doi.org/10.1158/1078-0432.CCR-16-2900.

42. Zhang, Z., Tan, X., Jiang, Z., Wang, H., and Yuan, H. (2022). Immune checkpoint inhibitors in osteosarcoma: A hopeful and challenging future. Front. Pharmacol. *13*, 1031527. https://doi.org/10.3389/fphar.2022.1031527.

43. Wu, C.C., Beird, H.C., Andrew Livingston, J., Advani, S., Mitra, A., Cao, S., Reuben, A., Ingram, D., Wang, W.L., Ju, Z., et al. (2020). Immuno-genomic landscape of osteosarcoma. Nat. Commun. *11*, 1008. https://doi.org/10.1038/s41467-020-14646-w.

44. Kajiwara, Y., Tazawa, H., Yamada, M., Kanaya, N., Fushimi, T., Kikuchi, S., Kuroda, S., Ohara, T., Noma, K., Yoshida, R., et al. (2023). Oncolytic virus-mediated reducing of myeloid-derived suppressor cells enhances the efficacy of PD-L1 blockade in gemcitabine-resistant pancreatic cancer. Cancer Immunol. Immunother. *72*, 1285–1300. https://doi.org/10.1007/s00262-022-03334-x.

45. Kusumi, T., Nishi, T., Tanaka, M., Tsuchida, S., and Kudo, H. (2001). A murine osteosarcoma cell line with a potential to develop ossification upon transplantation. Jpn. J. Cancer Res. *92*, 649–658. https://doi.org/10.1111/j.1349-7006.2001.tb01144.x.

46. Nishi, T., Kusumi, T., Tanaka, M., Sato, F., Sasaki, M., Kudo, H., and Kijima, H. (2008). Establishment of transplantable murine osteosarcoma cell line with endochondral ossification. Anticancer Res. *28*, 1627–1631.

47. Yamasaki, Y., Tazawa, H., Hashimoto, Y., Kojima, T., Kuroda, S., Yano, S., Yoshida, R., Uno, F., Mizuguchi, H., Ohtsuru, A., et al. (2012). A novel apoptotic mechanism of genetically engineered adenovirus-mediated tumour-specific p53 overexpression through E1A-dependent p21 and MDM2 suppression. Eur. J. Cancer *48*, 2282–2291. https://doi.org/10.1016/j.ejca.2011.12.020.

OMTON, Volume *32*

Supplemental information

p53-armed oncolytic virotherapy induces abscopal effect in osteosarcoma by promoting immunogenic cell death

Koji Demiya, Hiroshi Tazawa, Hiroya Kondo, Miho Kure, Yusuke Mochizuki, Tadashi Komatsubara, Aki Yoshida, Koji Uotani, Joe Hasei, Tomohiro Fujiwara, Toshiyuki Kunisada, Yasuo Urata, Shunsuke Kagawa, Toshifumi Ozaki, and Toshiyoshi Fujiwara

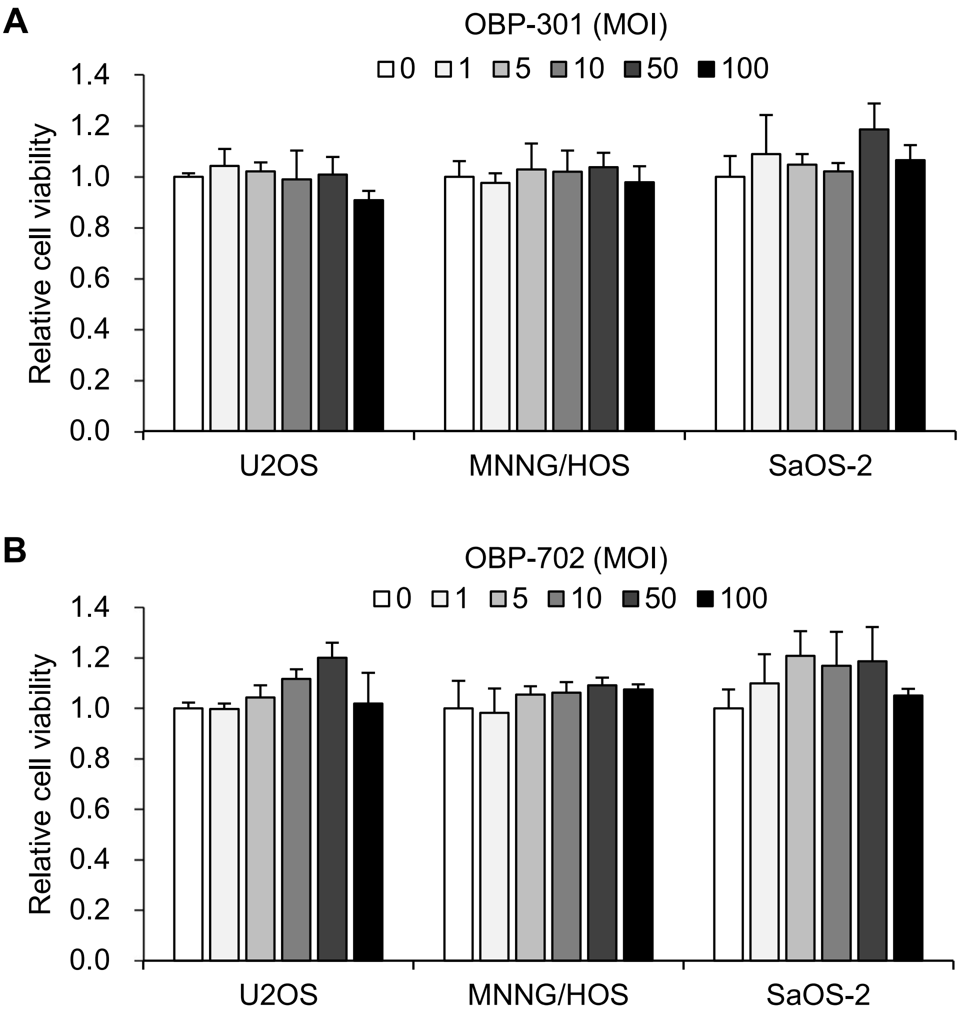

Figure S1. No cytopathic effect of OBP-301 and OBP-702 against human OS cells 24 h after infection. A,B U2OS, MNNG/HOS, and SaOS-2 cells were infected with OBP-301 (**A**) or OBP-702 (**B**) at the indicated multiplicity of infection (MOI), and cell viability was quantified 24 h after treatment using the XTT assay. Cell viability was calculated relative to that of the mock-infected group, which was set at 1.0. Cell viability data are expressed as mean values ±SD (n = 5).

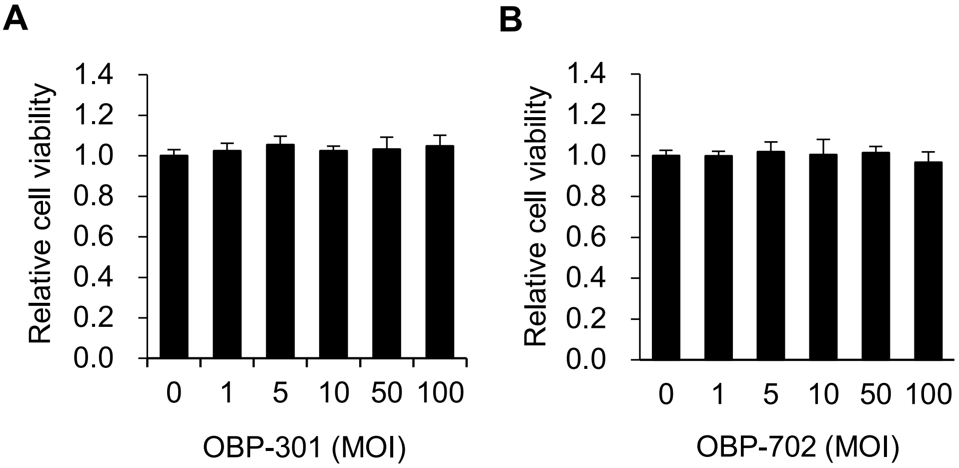

Figure S2. No cytopathic effect of OBP-301 and OBP-702 against murine OS cells 24 h after infection. A,B NHOS cells were infected with OBP-301 (**A**) or OBP-702 (**B**) at the indicated multiplicity of infection (MOI), and cell viability was quantified 24 h after treatment using the XTT assay. Cell viability was calculated relative to that of the mock-infected group, which was set at 1.0. Cell viability data are expressed as mean values ±SD (n = 5).

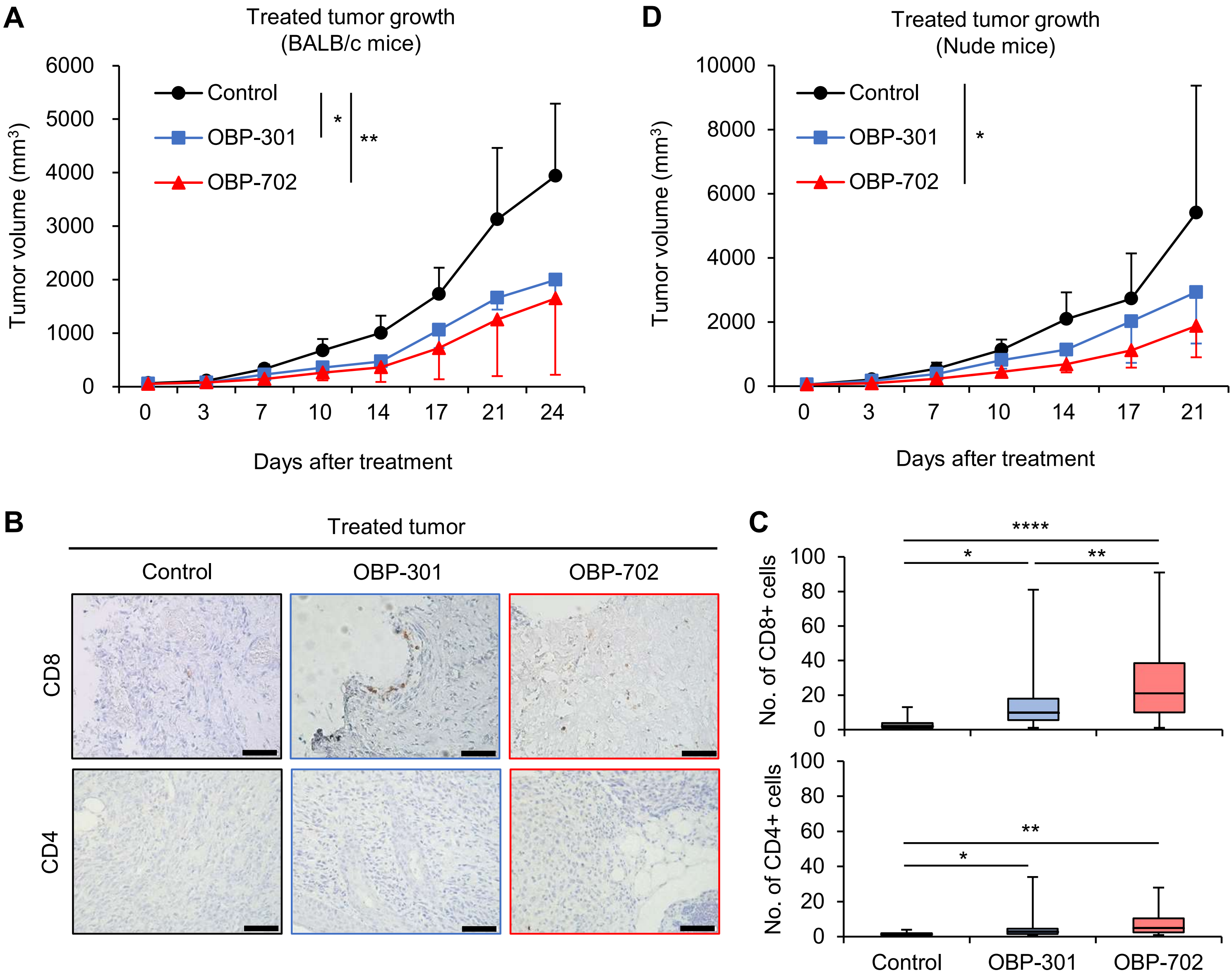

Figure S3. ***In vivo*** **antitumor effect of OBP-301 and OBP-702 in bilateral subcutaneous NHOS tumor models using immune-competent and immune-deficient mice.** **A** NHOS cells (2×10^6 cells/site) were inoculated into the bilateral flanks of immune-competent BALB/c mice. One side was intratumorally treated with PBS (n = 6), OBP-301 (blue arrows) (n = 7) or OBP-702 (red arrows) (n = 7) with 1×10^8 PFUs once a week for three cycles, and the other side was left untreated. The volume of NHOS tumors was monitored separately at the treated and untreated sites until day 24. **B** Representative photographs of immunohistochemical staining for CD8+ T cells and CD4+ T cells in treated tumors for each group. Scale bars, 100 μm. **C** The numbers of CD8+ T cells and CD4+ T cells were calculated from five different randomly selected fields. **D** The same experiment shown in **A** was performed using immune-deficient BALB/c-nu/nu nude mice, and tumor volume was monitored until day 21 (n = 8 in each group). Data are expressed as mean values ±SD. *$P < 0.05$, **$P < 0.01$, **** $P < 0.0001$.
